# Psychopharmacological effects and safety of styryl‐2‐pyrones and dihydrostyryl‐2‐pyrones‐rich fraction from *Polygala sabulosa*: absence of withdrawal syndrome and tolerance to anxiolytic‐like and anticonvulsant effects

**DOI:** 10.1111/jphp.12960

**Published:** 2018-06-28

**Authors:** Filipe Silveira Duarte, Marcelo Duzzioni, Rafael Luiz Prim, Alcíbia Maia Cardozo, Claudia Regina dos Santos, Maria Goretti da Silva, Maria Beatriz Cacese Shiozawa, Beatriz Garcia Mendes, Tiago Tizziani, Inês Maria Costa Brighente, Moacir Geraldo Pizzolatti, Thereza Christina Monteiro de Lima

**Affiliations:** ^1^ Department of Physiology and Pharmacology Federal University of Pernambuco Recife Pernambuco Brazil; ^2^ Institute of Biological Sciences and Health Federal University of Alagoas Maceió Alagoas Brazil; ^3^ Department of Pathology Federal University of Santa Catarina Florianópolis Santa Catarina Brazil; ^4^ Department of Chemistry Federal University of Santa Catarina Florianópolis Santa Catarina Brazil; ^5^ Department of Pharmacology Federal University of Santa Catarina Florianópolis Santa Catarina Brazil

**Keywords:** anxiety, convulsions, dihydrostyryl‐2‐pyrones, *Polygala sabulosa*, styryl‐2‐pyrones, tolerance, withdrawal symptoms

## Abstract

**Objectives:**

To investigate whether mice develop tolerance to the anxiolytic‐like and anticonvulsant effects of subchronic treatment with EA (the styryl‐2‐pyrones and dihydrostyryl‐2‐pyrones‐rich fraction of *Polygala sabulosa*), as well as any withdrawal symptoms after abrupt discontinuation; to compare the effects of EA with those of diazepam (DZP) on withdrawal‐induced anxiety; and to evaluate the toxicity of EA according to OECD guidelines.

**Methods:**

Male or female mice were acutely or subchronically treated with EA or DZP, and their tolerance to anxiolytic (evaluated in the elevated plus maze, EPM) and anticonvulsant effects (measured against pentylenetetrazole (PTZ)‐induced convulsions) were investigated. Other groups received EA or DZP for 28 days followed by withdrawal, being the anxiety‐like behaviour evaluated in the EPM.

**Key findings:**

Both acute and subchronic treatments with EA induced an anxiolytic effect in the EPM. The anticonvulsant activity of DZP, but not EA, was reduced by protracted treatment. EA withdrawal retained the anxiolytic profile, while DZP withdrawal induced anxiogenesis. EA counteracted the anxiogenic‐like actions of DZP withdrawal. EA has low toxicity as it did not cause any changes in the biochemical, haematological and histopathological markers.

**Conclusions:**

EA avoids the development of tolerance to its anxiolytic‐like and anticonvulsant actions, and does not promote withdrawal syndrome. EA does not cause relevant toxic effects in rodents.

## Introduction

Generalized anxiety disorder is a common chronic illness that has attracted considerable attention from scientists seeking to develop anxiolytic drugs with less side effects than existing drugs but that could maintain efficacy during chronic treatment.^1^, ^2^ Despite the clinical benefits of acute therapy with benzodiazepine (BDZ)‐type anxiolytics and its high therapeutic index, prolonged use of these drugs is associated with the development of tolerance. Furthermore, physical dependence among users is common leading to anxiety, aggressiveness and enhanced convulsive readiness (withdrawal syndrome) after abrupt cessation of treatment.^3^, ^4^

One of the most promising alternative sources for developing new anxiolytic medicines are traditional herbal remedies, the active compounds of which can be used in the development of standardized phytomedicines with proven efficacy in neuropsychiatry practice.^5^, ^6^, ^7^ Among such plants, *Passiflora incarnata L*., *Valeriana officinalis L and kava‐kava have* been extensively used as a folk medicine to treat anxiety. However, for the majority of them, the active principles are not yet well known.

*Polygala sabulosa* A. W. Bennett (Polygalaceae), popularly named ‘timutu‐pinheirinho’, grows abundantly in the Southern Meridional Highlands of Brazil and has been used in regional folk medicine as expectorant, topical anaesthetic and to treat disorders of the bowel and kidneys.^8^ Although its folk use is not related to psychopathologies, phytochemical studies of *P. sabulosa* isolated seven compounds – dihydrostyryl‐2‐pyrones (1, 2 or 3) and styryl‐2‐pyrones (4, 5, 6 or 7) – with a structure similar to the kavalactones found in *kava‐kava*, a folk medicine from Fiji Islands, Oceania, extensively used to treat anxiety.^9^, ^10^ Using a chemosystematic approach to select target plants in drug discovery, previously we investigated the psychopharmacological profile of this plant species in rodents and we had documented its acute anticonvulsant, hypnosedative and anxiolytic‐like properties. Furthermore, we identified the dihydrostyryl‐2‐pyrones and styryl‐2‐pyrones compounds as the only constituents of the ethyl acetate fraction obtained from *P. sabulosa* (hereafter referred to as EA).^11^ The medicinal effects observed were presumptively attributed to an interaction of these compounds with the BDZ binding site (BDZ‐bs),^12^ suggesting that *P. sabulosa* has potential for treating generalized anxiety and convulsions. However, to date, it is unknown whether the effectiveness of EA declines with usage (tolerance) or if its continued administration could induce withdrawal symptoms. Moreover, the toxicological potential of this plant had never been studied. Thus, this study was carried out to investigate the following points: (1) the putative development of tolerance (after a subchronic treatment regimen, 28 days) to the anxiolytic‐like (evaluated in the elevated plus maze test, EPM) and anticonvulsant (measured against pentylenetetrazole‐induced convulsions, PTZ) effects of EA, comparing those to the effects of diazepam (DZP), in male and female mice; (2) the putative development of withdrawal syndrome on abrupt cessation of treatment with EA or DZP in mice of both sexes evaluated in the EPM; (3) the ability of acute treatment with EA in counteracting the DZP withdrawal‐induced anxiety in mice evaluated in the EPM; (4) to evaluate the acute and subchronic (28 days) toxicity of EA treatment in rats and mice in accordance with Organization for Economic Co‐operation and Development (OECD) Guidelines 425^13^ and 407.^14^ Mortality, clinical signs, body mass gain, food and water intake, haematological and biochemical parameters, organ weights and histological markers were also monitored during this last step of the study.

## Material and methods

### Ethical statement

All experiments were conducted in accordance with international standards of animal welfare recommended by the Brazilian Society of Neuroscience and Behavior (Act 1992) and approved by the local Committee for Animal Care in Research (23080.027554/2004‐49/UFSC and 23076.013877/2015‐77/UFPE).

### Animals

Male and female adult Swiss mice (30–45 g) were used in the behavioural experiments and subchronic toxicity assays. Female adult Wistar rats (220–300 g) were used for the acute toxicity tests. All animals were maintained on a 12‐h light–dark cycle (lights on at 7:00 a.m.) at constant room temperature (23 ± 2 °C). They were grouped and housed in plastic cages and had free access to food and water, except during the experiments. All animals were allowed to adapt to laboratory conditions for at least 1 week before the behavioural assessments. We employed the minimum number of animals and duration of observations needed to obtain consistent data.

### Plant material and extracts

*Polygala sabulosa* was collected from Rancho Queimado (Santa Catarina State, Brazil, 27°51′32″S 49°07′41″W), in February 2003, at the same place where it was previously collected and identified by Prof. Dr. Olavo de Araújo Guimarães. A voucher specimen (#19640) was deposited in the Herbarium of the Department of Botany, Universidade Federal do Paraná (Curitiba, PR, Brazil). The procedures used to prepare the EA and to isolate its compounds have been described previously.^9^, ^10^ Briefly, a whole plant specimen was air‐dried and powered and compounds were extracted with 96% ethanol at room temperature. The crude extract, after removal of the solvent, was dissolved in methanol:H_2_O and partitioned with hexane and ethyl acetate successively to give hexane and ethyl acetate (EA) soluble fractions. The EA obtained were processed by flash chromatography or crystallization to afford the isolated compounds. Detailed NMR spectroscopic analysis (1H and 13C) of the EA and comparison of the physical and spectroscopic data with those reported established the structure of all compounds found in EA: 4‐methoxy‐6‐(11,12‐methylenedioxydihydrostyryl)‐2‐pyrone‐1 (DST‐1 = 1.87 mg/g), 4‐methoxy‐6‐(11,12‐methylenedioxy‐14‐methoxy‐dihydrostyryl)‐2‐pyrone‐2 (DST‐2 = 4.17 mg/g), 4‐methoxy‐6‐(11,12‐methylenedioxy‐10, 14‐dimethoxy‐dihydrostyryl)‐2‐pyrone‐3 (DST‐3 = 3.09 mg/g), 4‐methoxy‐6‐(11,12‐methylenedioxystyryl)‐2‐pyrone‐4 (STY‐4 = 3.60 mg/g), 4‐methoxy‐6‐(11,12‐methylenedioxy‐14‐methoxystyryl)‐2‐pyrone‐5 (STY‐5 = 3.67 mg/g), 4‐methoxy‐6‐(11,12‐methylenedioxy‐10,14‐dimethoxystyryl)‐2‐pyrone‐6 (STY‐6 = 5.61 mg/g) and 4‐methoxy‐6‐(11,12‐dimethoxystyryl)‐2‐pyrone‐7 (STY‐7 = 1.77 mg/g).^9^, ^10^

### Drugs and solvents

Diazepam (DZP, Dienpax^®^; Sanofi‐Winthrop Lab., São Paulo, SP, Brazil) was used as a reference drug. It was dissolved in saline (0.9% NaCl), while EA was freshly suspended in 10% Tween‐80 and tap water. Both drugs were always administered in a constant volume of 0.1 ml/10 g.

### Behavioural tests

#### Evaluation of development of tolerance to anxiolytic‐like effects

The putative development of tolerance to the anxiolytic‐like activity promoted by EA after a subchronic treatment was assessed using the elevated plus maze (EPM), as proposed by Lister.^15^ Male and female mice (*n* = 10–12/gender/treatment) received p.o. vehicle (10% Tween‐80 in water), EA (0.25–1 g/kg) or DZP (2 mg/kg) in an acute dose or in daily doses over 28 days. These treatment regimens are based on previous studies realized by other groups.^16^, ^17^, ^18^ One hour after the last administration, they were placed on the central platform, facing an enclosed arm, and observed for a 5‐min period. Frequencies of entries into either open or enclosed arms, as well as the time spent in each arm type, were recorded (in seconds). Subsequently, the percentage of time spent in the open arms (%TOA) was calculated. The number of entries into the enclosed arms (EEA) was used as an index of general activity.^19^ The incidence of ethological parameters such as unprotected head‐dipping (uHD) and protected stretch‐attend postures (pSAP) was also recorded.^11^, ^20^

#### Evaluation of development of tolerance to anticonvulsant effects

Male and female mice (*n* = 10/gender/treatment) received p.o. vehicle (10% Tween‐80 in water), EA (0.25–1 g/kg) or DZP (4 mg/kg) in an acute dose or in daily doses over 28 days. One hour after the last administration, all animals received pentylenetetrazole (PTZ 80 mg/kg, i.p.) and the latency (in s) and duration (in min) of the first convulsive episode (clonic or tonic/clonic convulsion) were recorded for up 30 min.

#### Evaluation of withdrawal syndrome

Male or female mice were orally pretreated on a daily basis with vehicle (10% Tween‐80 in water), EA (0.50 g/kg) or DZP (2 mg/kg) over 28 days, followed by no treatment for 3 days to induce withdrawal effects. Animals were then randomly divided into the following experimental groups 72 h after the last pretreatment: *Group 1* (*n* = 10/gender) – male or female mice receiving protracted pretreatment with vehicle that were acutely treated with vehicle; *Group 2* (*n* = 10/gender) – male or female mice receiving protracted pretreatment of DZP (2 mg/kg) that were acutely treated with vehicle; *Group 3* (*n* = 10/gender) – male or female mice receiving protracted pretreatment with DZP (2 mg/kg) that were acutely treated with DZP (2 mg/kg); *Group 4* (*n* = 10/gender) – male or female mice receiving protracted pretreatment with EA (0.50 g/kg) that were acutely treated with vehicle; *Group 5* (*n* = 10/gender) – male or female mice receiving protracted pretreatment with EA (0.50 g/kg) that were acutely treated with EA (0.50 g/kg); *Group 6* (*n* = 10/gender) – male or female mice receiving protracted pretreatment with DZP (2 mg/kg) that were acutely treated with EA (0.50 g/kg). Possible withdrawal effects in the form of anxiety‐like behaviour were recorded 72 h after withdrawal from the protracted treatment with DZP or EA were evaluated through the EPM test.

### Acute toxicity study

EA was administered by gavage at a dose of 2 g/kg to one adult female rat^13^ under an 8‐h period of fasting. We decided to start at a dose of 2 g/kg after the preliminary experiment. Sequentially, at intervals of 48 h, the same dose was given to four adult female rats, totalling five treated animals (EA 2 g/kg group). A control group was treated with the vehicle (10% Tween‐80 in water) to establish a comparative negative control group.^13^ Animals were periodically observed during the first 24 h after the administration of the EA and, thereafter, daily for 14 days. The putative acute toxicity of EA was investigated using the Hippocratic screening procedure suggested by Malone and Robichaud.^21^ Each group was periodically observed during the first 24 h after the administration of the EA and thereafter, daily for 14 days, and the number of animals that presented the following signs were registered: palpebral ptosis, hyperaemia, cyanosis, sialorrhea, altered respiratory frequency, piloerection, altered urinary volume, diarrhoea, circular movement, locomotor activity, aggressive behaviour, ataxia, loss of righting reflex, tail‐press response, paralysis of head and/or limbs, convulsions, abdominal contortions (writhes) and Straub's sign. During the 14 days of observation, the rats’ body weights, water and food intake were recorded daily.^13^ Mortality was registered and LD50 was estimated. At the end of the observation period, all animals were euthanized by isoflurane anaesthesia (inhalation) followed by exsanguination. Their organs (spleen, liver, kidneys, heart, lungs and brain) were removed, weighed and examined macroscopically and microscopically when adequate.

### Subchronic toxicity study

Male and female mice were divided into five experimental groups (*n* = 10 per gender per treatment groups). Three different doses of EA were established according to the LD50 obtained in the acute toxicity test: (1) EA group 0.25 g/kg; (2) EA group 0.50 g/kg, and; (3) EA group 1 g/kg. All EA doses were administered by gavage daily for 28 consecutive days. The negative control group received only the vehicle (10% Tween‐80 in water).^14^ Another group (denoted Satellite) received the maximum dose of 1 g/kg of the EA for 28 days and remained untreated for 14 additional days to observe reversibility, persistence or delayed occurrence of toxic effects related to administration of the extract. Each week, behaviour and physiological signs were observed and the animals were weighed (Bioprocess BS3000 model balance). Food and water consumption for groups of ten mice in two cages (five in each) were measured weekly. The food and water consumption was determined for each group, including spilled water and feed, and simply divided by five to calculate daily food (g/animal/day) and water (ml/animal/day) consumption.

On the final day of the experiment, mice were anaesthetized with ketamine (100 mg/kg; Vetbrands, São Paulo, SP, Brazil) and xylazine (20 mg/kg; Vetbrands, São Paulo, SP, Brazil), and after blood collection, they were sacrificed by an overdose of anaesthetic solution.

#### Biological, biochemical, anatomical and histological analysis

Blood samples were collected from the abdominal inferior vena cava with a syringe (1 ml). The first 300 μl sample from each mouse was collected in an *Expender* tube containing 0.1% ethylene diamine tetra‐acetic acid (EDTA), manually homogenized until the blood cell count. The remaining volume of blood was transferred to another *Eppendorf* tube (without anticoagulant) and 30 min later centrifuged (1500*g*, 15 min), with the serum being separated and stored for 1 day at +4 °C until the biochemical parameters were evaluated. Glucose, urea, creatinine, total proteins (TP), albumin (ALB), globulin (GLO), total cholesterol, triglycerides, alanine aminotransferase (ALT), alkaline phosphatase (ALP), aspartate aminotransferase (AST) and γ‐glutamyl aminotransferase (γ‐GT) were analysed using Cobas Mira automatic analyser (Roche Diagnostics, Indianapolis, Indiana, USA). Biochemical analyses were carried out from two different serum samples.

Red blood cells (RBC), leucocytes, blood count, mean corpuscular volume (MCV), haemoglobin and platelets were measured by an automatic analyser (Pentra 120; ABX Diagnostics, Montpellier, France). Leucocyte differentials (lymphocytes, monocytes, neutrophils and eosinophils) were determined through the May‐Grünwald‐Giemsa technique under a light microscope (BX41 microscope; Olympus, Tokyo, Japan).

All mice were autopsied for gross examination of the anatomical localization and visual changes in the organs. Selected vital organs (spleen, liver, kidneys, heart, lungs and brain) were carefully examined for macroscopic abnormalities, excised and trimmed of fat and connective tissue and then weighed (with the exception of the lungs). The organ‐to‐body weight ratio was calculated. Following conventional histological methods, organs were preserved in 10% neutral‐buffered formalin solution and embedded in paraffin, sectioned and stained with haematoxylin and eosin. In addition, cross sections (2‐μm‐thick) of liver from male and female mice treated with the vehicle or 1 g/kg of EA were analysed by optical microscopic and the following parameters were noted: architectural pattern; portals structures (vascular and biliar, presence of inflammatory cells); periportal activity (interface hepatitis); parenchymal changes (hepatocellular swelling, steatosis, necrosis and inflammatory infiltrate) and presence of pigments (biliary and hemosiderin). Each section was analysed by an experienced pathologist (blind to the treatments) who semi‐quantitatively scored changes as follows: 0 (absent or minimum), + (mild), ++ (moderate) and +++ (severe).

### Data analysis

All results are presented as mean ± SEM. The behavioural data were analysed by one‐way or two‐way (drug treatment × treatment time) ANOVA followed by the *post hoc* Student‐Newman‐Keuls' test for multiple comparisons. In the acute toxicity assay, Student's *t‐*test was used to compare the two groups, while for the subchronic toxicity assays, the differences between groups were determined by one‐way ANOVA followed by the *post hoc* Student‐Newman‐Keuls' test. Differences were considered statistically significant when *P* ≤ 0.05. All tests were performed using the Statistica^®^ software package (StatSoft Inc., Tulsa, OK, USA).

## Results

### Evaluation of development of tolerance to anxiolytic‐like effects

**Figure 1:**
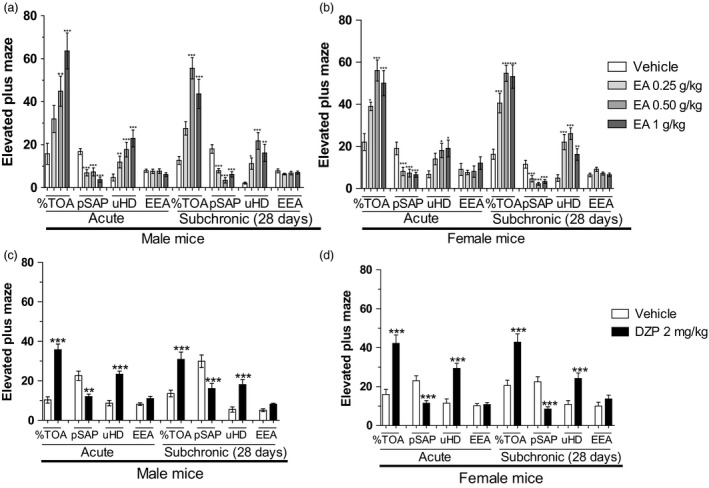
Effects of the oral acute or subchronic treatments (28 days) with the ethyl acetate fraction from *Polygala sabulosa* (EA, 0.25–1 g/kg) or diazepam (DZP, 2 mg/kg) on the behaviour of male (a,c) and female (b,d) mice evaluated in the EPM. Percentage of time spent in open arms (%TOA), number of unprotected head‐dipping movements (uHD), protected stretch‐attend postures (pSAP) and entries into the enclosed arms (EEA) are shown. Each value represents the mean ± SEM of 10–12 animals per group. *P ≤ 0.05, **P ≤ 0.01 and ***P ≤ 0.001 as compared to control group (vehicle) (two‐way ANOVA followed by Student‐Newman‐Keuls' test).

The effects of acute or subchronic treatments with EA or DZP on the main anxiety‐related parameters evaluated in the EPM are shown in Figure 1. Two‐way ANOVA failed to show any significant main effect for the treatment time factor, as well as any significant interaction between the treatments vs treatment time factors. However, in both male and female mice, there was a significant main effect for the drug treatment on the variables %TOA [male mice, drug treatment factor: *F*(7,88) = 21.82 and female mice, drug treatment factor: *F*(7,88) = 26.01, both *P* < 0.0001], pSAP [male mice, drug treatment factor: *F*(7,88) = 45.09 and female mice, drug treatment factor: *F*(7,88) = 43.26, both *P* < 0.0001] and uHD [male mice, drug treatment factor: *F*(7,88) = 15.75 and female mice, drug treatment factor: *F*(7,88) = 16.18, both *P* < 0.0001]. Student‐Newman‐Keuls' test showed that the acute treatment with EA (0.25–1 g/kg) increased the %TOA (*P* < 0.0001) and the amount of uHD (*P* < 0.0001) as well as reducing the number of pSAP (Figures 1a and 1b; all *P* < 0.0001). Male or female mice subchronically treated with EA showed a similar profile to that induced by acute treatment, with EA eliciting an increase in the %TOA and in the amount of uHD as well as a reduction in the number of pSAP (all *P* < 0.0001; Figure 1a and 1b).

The behavioural changes promoted by EA were similar to those induced by DZP in male and female mice, with a significant main effect for the drug treatment on the variables %TOA [male mice, drug treatment factor: *F*(3,36) = 68.52 and female mice, drug treatment factor: *F*(3,36) = 45.96, both *P* < 0.0001], pSAP [male mice, drug treatment factor: *F*(3,36) = 24.05 and female mice, drug treatment factor: *F*(3,36) = 39.08, both *P* < 0.0001] and uHD [male mice, drug treatment factor: *F*(3,36) = 59.36 and female mice, drug treatment factor: *F*(3,36) = 41.73, both *P* < 0.0001]. Student‐Newman‐Keuls' test showed that the acute or subchronic treatments with DZP increased the %TOA and the amount of uHD as well as reducing the number of pSAP (all *P* < 0.0001; Figures 1a and 1b).

The frequency of entries into enclosed arms was not significantly altered by any of the doses of EA or DZP in male and female mice (all *P* > 0.05; Figure 1a–1d).

### Evaluation of development of tolerance to anticonvulsant effects

**Figure 2:**
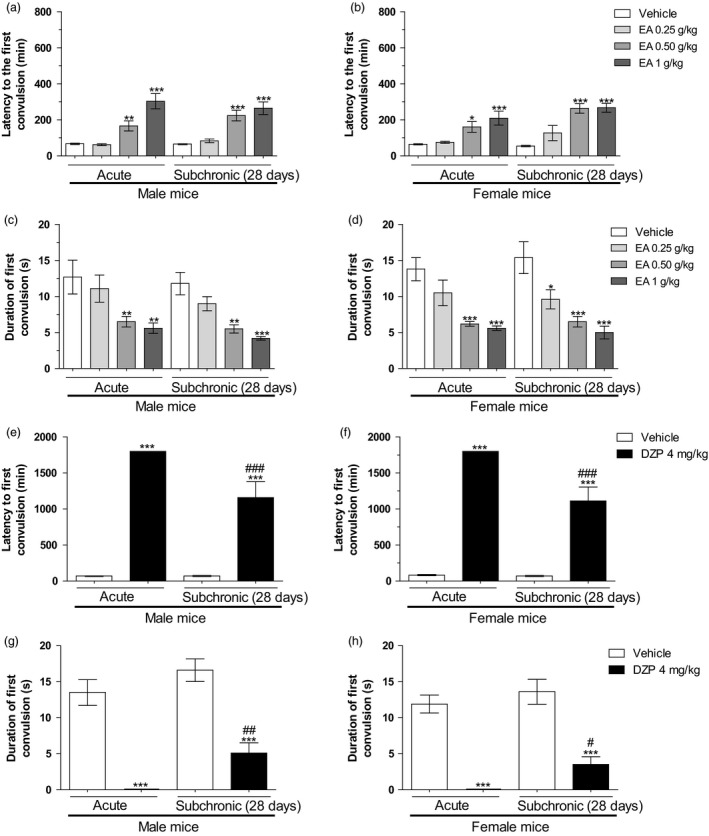
Effects of the oral acute or subchronic treatments (28 days) with the ethyl acetate fraction from *Polygala sabulosa* (EA, 0.25–1 g/kg) or diazepam (DZP, 4 mg/kg) on the latency to the first convulsive episode as well as its duration in male (a,c,e,g) and female (b,d,f,h) mice evaluated in the pentylenetetrazole (PTZ)‐induced convulsions test. Each value represents the mean ± SEM of 10 animals per group. *P ≤ 0.05, **P ≤ 0.01 and ***P ≤ 0.001 as compared to control group (vehicle); ^#^ *P* ≤ 0.05, ^##^ *P* ≤ 0.01 and ^###^ *P* ≤ 0.001 as compared to acute/DZP group (two‐way ANOVA followed by Student‐Newman‐Keuls' test).

**Figure 3:**
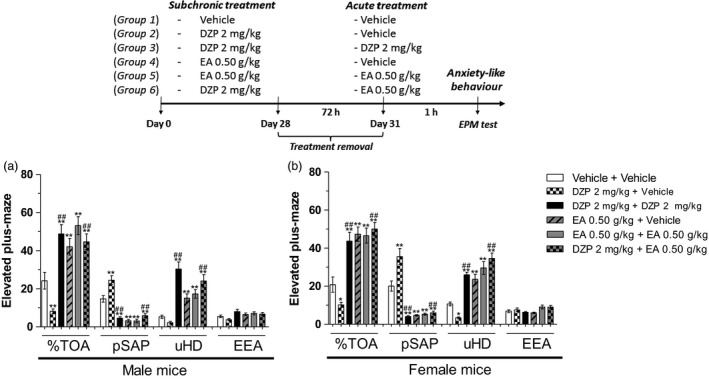
Effects of the oral acute or subchronic treatments (28 days) with the ethyl acetate fraction from *Polygala sabulosa* (EA, 0.50 g/kg) or diazepam (DZP, 2 mg/kg) on the main behavioural parameters evaluated in EPM test in male and female mice. In the top of figure, an outline of the treatments and experimental schedule summarizes the effects of the oral acute treatments with EA (0.50 g/kg) or DZP (2 mg/kg) on withdrawal syndrome induced by abrupt discontinuation of protracted treatments (28 days) with DZP in mice evaluated on the EPM. Each value represents the mean ± SEM of 10 animals per group. *P ≤ 0.05 and **P ≤ 0.01 as compared to control group (vehicle + vehicle); ^##^ *P* ≤ 0.01 as compared to DZP + vehicle or EA + vehicle groups (two‐way ANOVA followed by Student‐Newman‐Keuls' test).

Two‐way ANOVA did not detect any significant main effect for the treatment time factor, as well as any significant interaction between the treatments vs treatment time factors. However, in both male and female mice, there was a significant main effect for the drug treatment on the variables latency period to the first convulsive episode [male mice, drug treatment factor: *F*(7,72) = 35.50 and female mice, drug treatment factor: *F*(7,72) = 21.34, both *P* < 0.0001] and duration of the first convulsion [male mice, drug treatment factor: *F*(7,72) = 13.56 and female mice, drug treatment factor: *F*(7,72) = 20.48, both *P* < 0.0001]. Student‐Newman‐Keuls' test showed that the acute treatment with EA (0.50 or 1 g/kg) significantly increased the latency period to the first convulsive episode and reduced the duration of the first convulsion in both male and female mice (*P* < 0.0001). Animals subchronically treated with EA (0.25–1 g/kg) showed increased latency to the first convulsive episode and shorter duration of the first convulsion in mice of both sexes (*P* < 0.0001), in a similar way to mice acutely treated with EA (Figure 3a–3d). There was no statistical difference between the acute and subchronic EA treatments in male (Figure 2a and 2c; *P* > 0.05) and female mice (Figure 2b and 2d; *P* > 0.05).

Concerning the effects of DZP on the PTZ test, two‐way ANOVA detected a significant main effect for the drug treatment and treatment time factors, as well as interaction between the treatments vs treatment time factors on the latency to the first convulsive episode [male mice, drug treatment factor: *F*(3,36) = 163.14, *P <* 0.0001; treatment time factor: *F*(3,36) = 8.39, *P <* 0.001; interaction factor: *F*(3,36) = 8.49, *P <* 0.0001; female mice, drug treatment factor: *F*(3,36) = 192.04, *P <* 0.0001; treatment time factor: *F*(3,36) = 12.35, *P <* 0.001; interaction factor: *F*(3,36) = 11.54, *P <* 0.001]. An anticonvulsant effect was observed after the subchronic treatment with DZP; however, the magnitude of DZP effects on the latency to the first convulsive episode was significantly smaller than those of acute treatment in mice of both sexes (Figure 2e–2f; both *P <* 0.0001). In relation to duration of the first convulsion, two‐way ANOVA detected a significant main effect for the drug treatment and treatment time factors in both sexes [male mice, drug treatment factor: *F*(3,36) = 80.84, *P <* 0.0001; treatment time factor: *F*(3,36) = 8.70, *P <* 0.01; female mice, drug treatment factor: *F*(3,36) = 84.12, *P <* 0.0001; treatment time factor: *F*(3,36) = 4.70, *P <* 0.05], but did not detect any significant interaction between the two factors in male and female mice (*P >* 0.05). Student‐Newman‐Keuls' test showed that there was a statistical difference between the acute and subchronic DZP treatments on the duration of the first convulsion in male and female mice (Figure 2g–2h; *P <* 0.01 and *P ≤* 0.05, respectively).

### Evaluation of withdrawal syndrome

Figure 3 shows the behavioural changes after the abrupt discontinuation of subchronic treatment (28 days) with DZP or EA in male and female mice evaluated in the EPM. In EA (0.50 g/kg)‐treated mice group, EA withdrawal after 72 h retained its anxiolytic‐like profile of action, as revealed by increase in the %TOA [male mice: *F*(5,54) = 17.33 and female mice: *F*(5,54) = 20.41, both *P* < 0.0001] and the amount of uHD [male mice: *F*(5,54) = 19.05 and female mice: *F*(5,54) = 28.77, both *P* < 0.0001] as well as reducing the number of pSAP [male mice: *F*(5,54) = 37.70 and female mice: *F*(5,54) = 37.40, both *P* < 0.0001] (Figures 3a and 3b, respectively). In DZP (2 mg/kg)‐treated mice group, DZP withdrawal after 72 h reduced the %TOA in male and female mice (both *P* < 0.0001) and the amount of uHD in female mice (*P* < 0.0001), as well as increased pSAP in both sexes (both *P* < 0.0001; Figures 3a and 3b, respectively). The acute administration of DZP 2 mg/kg or EA 0.50 g/kg was effective in counteracting the observed reduction in %TOA and uHD in male and female mice after abrupt DZP withdrawal (both *P* < 0.0001). Moreover, acute treatments with DZP or EA counteracted the observed increase in pSAP (both *P* < 0.0001). These effects were not accompanied by any alterations in the frequency of entries into enclosed arms (*P* > 0.05; Figure 3a and 3b).

### Acute and subchronic toxicity assays of EA

In the acute oral treatment, EA 2 g/kg induced palpebral ptosis and a reduction in locomotor activity in female rats. The oral LD_50_ was therefore considered to be above 2 g/kg. EA did not elicit any significant change in weight gain, food and water intake of female rats during the 14 days of observation period (*P* > 0.05; Table 1). There was no statistical difference in the relative weights of spleens, kidneys, hearts and brains of female rats acutely treated with EA 2 g/kg in relation to control group or macroscopic changes that indicate toxicity in any organs evaluated at the end of this experimental period (*P* > 0.05*;* Table 2).

**Table 1:** Body weight gain, food and water intake of rats observed for 14 days after a single oral treatment with the ethyl acetate fraction from *Polygala sabulosa* (EA, 2 g/kg) in an acute toxicity study and mice treated orally for 28 days with EA 0.25–1 g/kg in subchronic toxicity assay. Satellite group received EA 1 g/kg by 28 days and remained untreated for more 14 days

	Acute toxicity in rats				Subchronic toxicity in mice		
	Vehicle (*n* = 5)	EA 2 g/kg (*n* = 5)	Vehicle (*n* = 10)	EA 0.25 g/kg (*n* = 10)	EA 0.50 g/kg (*n* = 10)	EA 1 g/kg (*n* = 10)	Satellite (*n* = 10)
Female							
Initial weight (g)	210.00 ± 6.12	209.00 ± 6.96	36.32 ± 0.88	35.86 ± 0.97	34.08 ± 0.52	34.44 ± 1.06	36.00 ± 1.18
Final weight (g)	231.60 ± 3.88	232.00 ± 5.97	38.11 ± 0.76	38.38 ± 0.74	36.29 ± 0.49	36.84 ± 0.88	38.30 ± 1.23
Body weight gain (%)	10.59 ± 3.24	11.20 ± 2.37	6.62 ± 1.50	6.63 ± 1.42	6.02 ± 1.22	6.54 ± 1.74	6.71 ± 0.71
Food intake (g/day)	190.73 ± 5.51	192.61 ± 4.88	5.26 ± 0.59	6.32 ± 0.10	6.30 ± 0.12	6.26 ± 0.32	6.77 ± 0.14
Water intake (ml/day)	214.77 ± 4.75	212.84 ± 2.22	4.88 ± 0.2	5.32 ± 0.08	4.92 ± 0.03	5.03 ± 0.14	4.95 ± 0.13
Male							
Initial weight (g)	–	–	39.16 ± 0.67	37.03 ± 0.93	38.52 ± 0.73	37.96 ± 0.66	36.6 ± 0.56
Final weight (g)	–	–	42.67 ± 0.63	39.54 ± 0.93	41.90 ± 0.44	41.22 ± 0.69	39.2 ± 0.55
Body weight gain (%)	–	–	8.20 ± 1.00	6.35 ± 1.19	8.01 ± 1.70	7.74 ± 1.49	7.14 ± 0.64
Food intake (g/day)	–	–	6.62 ± 0.08	6.90 ± 0.10	7.13 ± 0.08	7.26 ± 0.11	7.00 ± 0.13
Water intake (ml/day)	–	–	5.66 ± 0.22	6.11 ± 0.04	6.26 ± 0.06	6.13 ± 0.1	5.87 ± 0.06

Data are expressed as the mean ± SEM. Data were analysed by Student's *t‐*test or one‐way ANOVA followed by Student‐Newman‐Keuls' test

**Table 2:** Relative organ weights (g/100 g of body weight) of rats observed for 14 days after a single oral treatment with the ethyl acetate fraction from *Polygala sabulosa* (EA, 2 g/kg) in an acute toxicity study; relative organ weights (g/10 g of body weight) of mice orally treated for 28 days with EA 0.25–1 g/Kg in subchronic toxicity assay. Satellite mice group received EA 1 g/kg by 28 days and remained untreated for more 14 days

	Acute toxicity in rats				Subchronic toxicity in mice		
	Vehicle (*n* = 5)	EA 2 g/kg (*n* = 5)	Vehicle (*n* = 10)	EA 0.25 g/kg (*n* = 10)	EA 0.50 g/kg (*n* = 10)	EA 1 g/kg (*n* = 10)	Satellite (*n* = 10)
Female							
Spleen	0.28 ± 0.03	0.26 ± 0.02	0.45 ± 0.02	0.42 ± 0.03	0.42 ± 0.04	0.43 ± 0.02	0.47 ± 0.04
Liver	4.85 ± 0.21	4.60 ± 0.15	4.48 ± 0.20	4.34 ± 0.17	4.43 ± 0.16	5.29 ± 0.17**	4.53 ± 0.25
Kidney	0.56 ± 0.04	0.54 ± 0.03	0.50 ± 0.02	0.51 ± 0.01	0.48 ± 0.02	0.48 ± 0.02	0.53 ± 0.03
Heart	0.53 ± 0.05	0.52 ± 0.04	0.35 ± 0.01	0.35 ± 0.01	0.34 ± 0.01	0.35 ± 0.01	0.37 ± 0.02
Brain	1.62 ± 0.16	1.54 ± 0.13	0.92 ± 0.01	0.89 ± 0.02	0.93 ± 0.02	0.92 ± 0.02	0.90 ± 0.01
Male							
Spleen	–	–	0.37 ± 0.02	0.33 ± 0.01	0.35 ± 0.03	0.31 ± 0.02	0.34 ± 0.03
Liver	‐	‐	4.37 ± 0.16	4.79 ± 0.18	4.81 ± 0.12	5.87 ± 0.17**	4.86 ± 0.15
Right kidney	–	–	0.56 ± 0.02	0.58 ± 0.02	0.59 ± 0.02	0.59 ± 0.01	0.58 ± 0.02
Heart	–	–	0.35 ± 0.01	0.37 ± 0.02	0.38 ± 0.02	0.37 ± 0.01	0.37 ± 0.01
Brain	‐	‐	0.76 ± 0.02	0.79 ± 0.02	0.81 ± 0.02	0.75 ± 0.02	0.79 ± 0.03

Data are expressed as the mean ± SEM. Data were analysed by Student's *t*‐test or one‐way ANOVA followed by Student‐Newman‐Keuls' test. **P < 0.01 as compared to respective control group (vehicle).

No deaths or adverse effects were observed in any groups of both sexes subchronically treated with the vehicle or EA (0.25–1 g/kg). One‐way ANOVA revealed no significant effect of treatments on body weight gain, food and water intake (*P* > 0.05; Table 1). At the end of the 28‐day observation period, no significant differences were observed in the relative weights of spleens, kidneys, hearts and brains of male or female mice treated with EA at doses of 0.25 or 0.50 g/kg (*P* > 0.05; Table 2). However, the dose of 1 g/kg significantly increased the relative liver weight in mice of both sexes [male mice: *F*(3,36) = 20.79 and female mice: *F*(3,36) = 11,89, both *P* < 0.001; Table 2]. Nevertheless, no delayed occurrence of toxic effects was observed in female and male mice of satellite group during all observation period as can be seen in the parameters weight gain, food and water intake (*P* > 0.05; Table 1), as well as in relative weights of main vital organs (*P* > 0.05*;* Table 2).

There was no relevant difference in biochemical and haematological parameters in the male and female mice treated with EA (0.25–1 g/kg) when compared to the control animals (*P* > 0.05; Table 3). EA 1 g/kg did not induce any significant changes in biochemical and haematological parameters in female and male mice of satellite group (*P* > 0.05; Table 3).

**Table 3:** Effect of the subchronic treatment (28 days) with the ethyl acetate fraction from *Polygala sabulosa* (EA, 0.25–1 g/kg) on biochemical and haematological parameters in male and female mice

Biochemical	Male mice					Female mice				
	Vehicle	EA 0.25 g/kg	EA 0.50 g/kg	EA 1 g/kg	Satellite	Vehicle	EA 0.25 g/kg	EA 0.50 g/kg	EA 1 g/kg	Satellite
	*n* = 5	*n* = 5	*n* = 5	*n* = 5	*n* = 5	*n* = 5	*n* = 5	*n* = 5	*n* = 5	*n* = 5
Creat (mg/dl)	0.45 ± 0.02	0.46 ± 0.03	0.39 ± 0.01	0.40 ± 0.04	0.32 ± 0.04	0.20 ± 0.03	0.17 ± 0.02	0.19 ± 0.03	0.17 ± 0.02	0.22 ± 0.02
Urea (mg/dl)	47.54 ± 4.17	46.22 ± 2.40	50.13 ± 2.02	53.05 ± 1.90	52.60 ± 3.40	48.28 ± 2.39	50.28 ± 1.38	45.80 ± 1.11	42.68 ± 2.68	50.40 ± 3.56
ALP (U/L)	155.00 ± 15.80	142.00 ± 15.60	148.17 ± 14.50	137.67 ± 21.40	130.60 ± 10.55	116.50 ± 9.66	112.17 ± 12.63	152.17 ± 15.42	134.83 ± 14.10	117.40 ± 7.60
γGT (U/L)	1.20 ± 0.20	1.40 ± 0.40	1.17 ± 0.17	1.33 ± 0.21	1.00 ± 0.16	1.17 ± 0.17	1.00 ± 0.00	1.00 ± 0.00	1.00 ± 0.00	1.00 ± 0.00
AST (U/L)	49.00 ± 3.56	60.4 ± 4.76	58.67 ± 5.11	74.83 ± 10.34	58.00 ± 4.06	60.17 ± 3.24	54.67 ± 2.89	63.67 ± 2.78	64.83 ± 4.08	60.60 ± 2.52
ALT (U/L)	41.02 ± 4.86	42.80 ± 3.76	37.00 ± 5.20	49.12 ± 4.78	49.40 ± 2.20	43.50 ± 3.35	35.67 ± 1.89	40.33 ± 1.45	45.33 ± 5.29	46.40 ± 2.64
TP (g/dl)	5.70 ± 0.16	5.48 ± 0.08	5.90 ± 0.07	6.08 ± 0.10	5.16 ± 0.22	5.53 ± 0.08	5.60 ± 0.10	5.78 ± 0.11	5.70 ± 0.16	5.08 ± 0.11
Alb (g/dl)	1.70 ± 0.05	1.74 ± 0.04	1.70 ± 0.03	1.68 ± 0.07	1.26 ± 0.08	1.68 ± 0.03	1.75 ± 0.03	1.78 ± 0.04	1.72 ± 0.07	1.26 ± 0.11
Glo (g/dl)	4.06 ± 0.12	4.10 ± 0.08	4.20 ± 0.08	4.40 ± 0.09	4.00 ± 0.22	3.85 ± 0.06	3.85 ± 0.11	4.00 ± 0.10	3.98 ± 0.11	3.60 ± 0.14

Haematological	*n* = 10	*n* = 10	*n* = 10	*n* = 10	*n* = 10	*n* = 10	*n* = 10	*n* = 10	*n* = 10	*n* = 10
RBC (10^6^/mm^3^)	8.79 ± 0.24	8.31 ± 0.27	8.06 ± 0.12	8.23 ± 0.16	8.02 ± 0.17	8.28 ± 0.18	8.31 ± 0.12	8.55 ± 0.12	8.10 ± 0.21	7.95 ± 0.20
HCT (%)	40.97 ± 1.17	42.,35 ± 1.63	39.90 ± 0.50	40.67 ± 0.87	38.88 ± 0.42	38.74 ± 0.99	40.81 ± 0.82	39.90 ± 0.65	38.49 ± 0.71	39.26 ± 0.48
HGB (g/dl)	14.00 ± 0.41	13.31 ± 0.41	12.93 ± 0.18	13.24 ± 0.33	12.36 ± 0.13	13.43 ± 0.30	13.45 ± 0.20	13.81 ± 0.22	13.41 ± 0.23	12.69 ± 0.0.23
MCV (μm^3^)	46.60 ± 0.54	48.88 ± 1.40	49.54 ± 0.37	49.45 ± 0.80	46.80 ± 0.25	46.64 ± 0.34	49.00 ± 0.57	46.64 ± 0.43	47.80 ± 0.68	47.20 ± 0.36
Leu (10^3^/mm^3^)	7.57 ± 0.51	5.04 ± 0.68	5.31 ± 0.91	5.56 ± 0.62	6.36 ± 0.37	5.65 ± 0.56	4.78 ± 0.44	5.42 ± 0.31	6.26 ± 0.55	7.04 ± 0.42
Neu (%)	4.60 ± 0.40	4.70 ± 0.58	6.15 ± 1.03	4.09 ± 0.69	5.30 ± 0.34	4.68 ± 0.71	6.22 ± 0.71	4.37 ± 0.64	4.60 ± 0.62	6.00 ± 0.47
Lym (%)	94.00 ± 0.47	91.90 ± 0.89	90.36 ± 1.52	92.54 ± 1.35	88.70 ± 1.92	94.82 ± 0.57	82.27 ± 1.10	94.27 ± 0.95	94.10 ± 0.71	87.70 ± 1.97
Mon (%)	2.10 ± 0.76	3.40 ± 0.76	2.73 ± 0.36	3.27 ± 1.10	2.40 ± 0.34	1.45 ± 0.47	2.73 ± 0.66	1.36 ± 0.34	1.20 ± 0.20	2.60 ± 0.43
Eos (%)	0.10 ± 0.10	0.00 ± 0.00	0.36 ± 0.20	0.09 ± 0.09	0.40 ± 0.16	0.00 ± 0.00	0.09 ± 0.09	0.18 ± 0.18	0.10 ± 0.10	0.50 ± 0.17
Plat (10^3^/mm^3^)	678.80 ± 65.34	874.30 ± 61.25	855.82 ± 36.80	817.36 ± 60.13	672.80 ± 39.34	703.64 ± 17.10	702.18 ± 35.00	638.36 ± 27.20	629.60 ± 23.13	697.40 ± 28.51

ALT, alanine aminotransferase; AST, aspartate aminotransferase; Alb, Albumin; ALP, alkaline phosphatase; Creat, creatinine; Eos, eosinophils; HCT, haematocrit; HGB, haemoglobin; γGT, γ‐glutamyl aminotransferase; Glo, globulin; Leu, leucocytes; Lyn, lymphocytes; MCV, mean corpuscular volume; Mon, monocytes; Neu, neutrophils;RBC, red blood cells; Plat, platelets. Data are expressed as the mean ± SEM of the biochemical (*n* = 5) and haematological (*n* = 10) parameters of male and female mice. Data were analysed by one‐way ANOVA followed by Student‐Newman‐Keuls' test.

**Figure 4:**
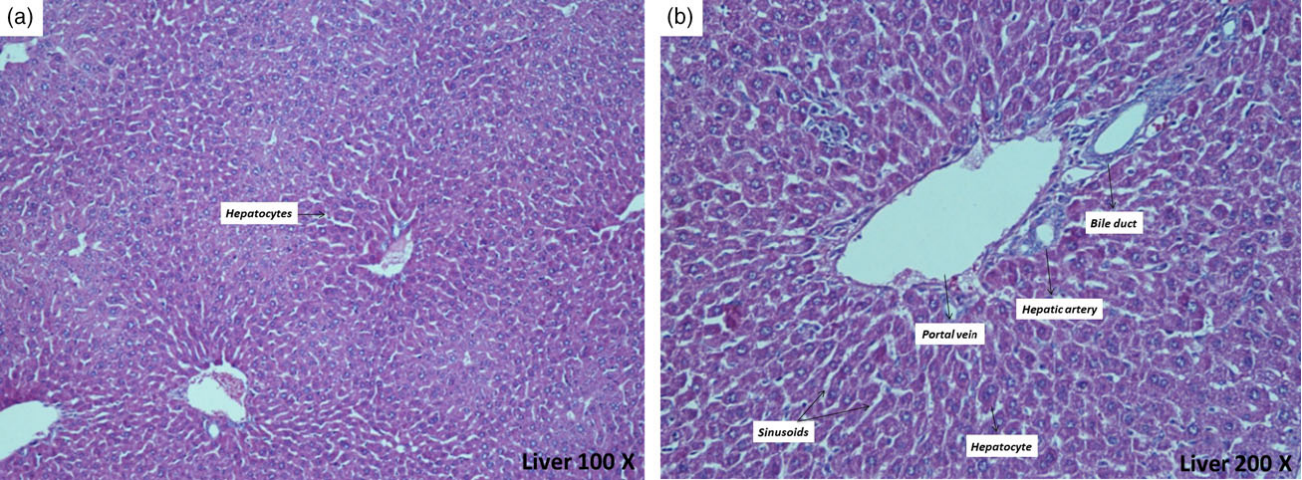
Photomicrographs of the liver from mice treated (28 days) with ethyl acetate fraction of *Polygala sabulosa* (EA, 1 g/kg). Transverse sections were stained with haematoxylin and eosin and observed at two different levels of magnification. (a) transverse sections (100×) showing lobular architecture totally conserved and (b) transverse sections (200×) showing hepatocytes maintained in a trabecular arrangement, vascular and biliary structures conserved and intact. [Colour figure can be viewed at http://wileyonlinelibrary.com]

No macroscopic and histological abnormality was observed in the organs examined (spleen, liver, kidneys, heart, lungs and brain) of control and experimental groups (Figures 4a and 4b show representative photomicrographs of the liver from male mice treated with EA 1 g/kg for 28 days). Histological analysis did not detect any deposits, pigments, significant structural changes or necro‐inflammatory injuries. The histological profile of liver cross sections was similar between control and EA (1 g/kg)‐treated animals of both sexes, with a normal liver architecture: hepatocytes well arranged, sinusoid capillaries and central veins without alterations.

## Discussion

In the present work, we greatly extended previous data by providing the first report of the anxiolytic‐like effects and safety of EA from *P. sabulosa* after subchronic treatment regimen. Specifically, we investigated a putative development of tolerance to the anxiolytic‐like and anticonvulsant effects of EA in male and female mice and compared these results with those of DZP. Moreover, the withdrawal syndrome was evaluated by assessing anxiety‐like behaviour in mice using the EPM, one of the most widely used well‐validated animal models of anxiety.^22^ Additionally, we also evaluated the acute and subchronic toxicity of EA in rats and mice according to the OECD in order to determine its safety.^13^, ^14^

Plant bioresources are relied upon as natural and inexpensive remedies for the management of several diseases; however, the growing interest aiming to maximize the potential of phytochemicals has led to the development of ‘rich fractions’, in which extracts contain bioactive compounds in addition to elevated levels of the primary compound.^23^ In relation to *P. sabulosa*, more recently we focused our studies on the EA because among the three plant fractions tested, only EA showed psychopharmacological effects in rodents.^11^ Subsequently, we attributed partial BDZ‐like activity to treatment with EA and several naturally occurring styrylpyrones isolated from *P. sabulosa*, through *in vivo* and *in vitro* experiments. Our *in vivo* findings showed that DST (1 and 3) and STY (4 and 7), centrally administered, elicited anxiolytic‐like effects in the range of doses from fmol to pmol, in an order of magnitude lower than those found for DZP (nmol). Compound (1) showed a less‐evident effect, whereas DST (3) and STY (7) were the most potent compounds, with effects similar to the DZP, while the compounds DST (2) and STY (5) were inactive in the EPM test.^11^ In the *in vitro* saturation experiments, the ability of DST (3) or STY (7) to modify the *K*_d_ without any significant change in the *B*_max_ suggests that these compounds interact competitively at only one recognition site, probably the BDZ‐bs.^12^ These results support the involvement of the BDZ‐bs of the GABA_A_ receptor in underlying the anticonvulsant and anxiolytic‐like actions of the EA as well as of DST and STY compounds isolated from *P. sabulosa*.

It is important to mention that EA is a complex mixture of seven chemical constituents, any number of which may be responsible for its psychopharmacological properties, and it is possible that a synergism of all compounds acting at the BZD‐bs may occur. Notwithstanding, more research is needed on all of these compounds individually and collectively. As these secondary metabolites are associated with an anxiolytic‐like effect, it is reasonable to extrapolate that EA containing all of the compounds may be effective in treating generalized anxiety and convulsions. However, the efficacy of such treatments would partly depend on whether the anxiolytic‐like and anticonvulsant effects induced by EA declined after protracted administration.

Studies have demonstrated the development of the tolerance to the hypnosedative and anticonvulsant properties of BDZ drugs.^24^, ^25^, ^26^ Nevertheless, data demonstrating tolerance to the anxiolytic‐like of these compounds have been inconsistent^27^, ^28^ and efficacy of repeated treatments remains a subject of debate.^24^, ^25^, ^26^ The reason for this discrepancy is unclear, but it may be due to differences in dosing regimen or the animal model used in the study.^29^, ^30^ The development of tolerance to the anxiolytic effects after long‐term use of BDZ has not been conclusively studied in humans.^31^ BDZ tolerance, if it occurs, would constitute an adaptive mechanism to chronic treatment through the mechanism of neuronal plasticity, subsensitivity of GABA_A_ receptors, reduced coupling between the BDZ agonist site and the chloride channel or change in the expression of GABA_A_ receptor subunits.^32^, ^33^, ^34^, ^35^ It should be noted that the excitatory glutamate system has also been implicated in the development of BDZ tolerance.^32^ In the current work, both acute and subchronic exposure resulted in an increase in the exploration of open arms of EPM in both male and female mice, indicating an anxiolytic‐like profile of action for EA. For the first time, our findings critically show that tolerance to the anxiolytic‐like effect of EA, it is not developed after subchronic treatment. Similarly, the development of tolerance to the anxiolytic effects of DZP following subchronic exposure was not observed. Studies show that a longer period of treatment together with the right dose of a drug is, in some cases, required to induce insensitivity.^29^, ^36^ Accordingly, tolerance to the anxiolytic‐like effects of DZP was observed in animals treated with low daily doses of DZP for 21 days. However, animals treated with a high daily dose of DZP for 21 days did not develop any tolerance to DZP‐induced anxiolytic effects.^29^ This finding is concordant with the results of our current study where both acute and chronic DZP‐treated animals showed similar anxiety‐related behaviours following 28 days of DZP treatment.

Studies in primates and rodents have demonstrated that protracted treatment with DZP led to the development of tolerance to its anticonvulsant actions.^37^, ^38^, ^39^, ^40^, ^41^ Here, we compared the tolerance liability of EA with that of DZP with respect to their protective effects against PTZ‐induced convulsions. We showed that male and female mice subchronically treated with DZP exhibited significant tolerance to anticonvulsant effects, as seen by reduced latency to the first PTZ‐induced convulsion. Moreover, the magnitude of DZP protective effects against the duration of first convulsive episode was significantly smaller when compared to the acutely treated group. In contrast, EA treatment did not lead to tolerance, still eliciting strong anticonvulsant and anxiolytic‐like properties. As EA is a fraction rich in several compounds with moderate affinity for the BDZ‐bs (μm) in comparison with DZP (nm),^12^ it is possible that in addition to its action on the GABA_A_ receptor, there may be other underlying mechanisms of action. These may contribute to the central effects and lack of tolerance (in contrast to BDZ compounds), and it could be possibly mediated through the participation of other ion channels and/or neurotransmitter systems. On this regard, previously we demonstrated that EA also protected mice against maximal electroshock‐induced convulsions in a similar way to several drugs that inhibit voltage‐dependent sodium channels or drugs that block glutamatergic excitation mediated by the NMDA receptor.^12^ This profile of action for EA is distinct from DZP, that only promoted a weak reduction in the hindlimb tonic extension in the MES test, and at doses considered ataxic and hypnotic.^12^ These findings are in agreement with the weak ability of BDZ drugs to prevent electroshock‐induced seizures at doses lower than the neurotoxic TD50.^42^, ^43^

Abrupt discontinuation of chronic treatment with BDZ drugs can cause withdrawal symptoms, especially increased anxiety.^44^, ^45^ Anxiety‐like behaviour after BDZ withdrawal in rats is commonly seen in the EPM 12–96 h after abrupt drug discontinuation.^46^, ^47^, ^48^ Moreover, an increased susceptibility to seizures, loss of body weight and decrease in food consumption has also been observed in mice and rats after abrupt discontinuation of repeated treatment with DZP.^49^, ^50^ In the present work, an anxiogenic‐like effect was observed 72 h after abrupt cessation of a protracted treatment with DZP. Protracted treatment with BDZ drugs may also induce excitatory signalling via glutamatergic system as part of a compensatory effect. Thus, it is possible that an overactive glutamatergic system (which may be associated with alterations in NMDA receptor subunit expression or changes in binding to this receptor) may be responsible for the symptoms of BDZ withdrawal.^18^, ^32^, ^51^

In contrast, EA treatment did not lead to any anxiogenic‐like behaviour after its withdrawal. Moreover, the rebound of anxiety induced by DZP withdrawal was blocked by the administration of EA – similar to that achieved by administration of DZP. And this finding suggests that EA treatment may be useful to reduce and/or prevent withdrawal syndrome caused by abrupt cessation to DZP.

One of the major problems in the use of traditional medicinal preparations is the lack of the scientific and clinical data about the effectiveness and the safety of these mixtures. Several plants have been extensively used as folk medicines to treat anxiety, in particular *kava‐kava*. However, hepatotoxicity has been linked to the consumption of kava extracts in Western countries,^52^, ^53^, ^54^, ^55^, ^56^, ^57^ although the mechanism of toxicity has not been formally established.^58^, ^59^ This is the reason that we decided in the present work to investigate the possible toxic effects of acute and subchronic treatment with EA as the compounds of EA are structurally related to the kavalactones. Consequently, we observed no deaths or, indeed, any changes in weight, food and water intake in female rats treated with a single dose of EA 2 g/kg. However, we did observe some putative toxic symptoms, such as a reduction in locomotor activity and palpebral ptosis. It should be noted that these signals may be a result of a BDZ‐like action^12^ and they are common to these drugs. A necropsy at the end of the experimental period revealed no gross changes in any organs. As the oral LD50 of EA for female rats is higher than 2 g/kg, EA can be classified as low toxicity and falls into Class 5.^13^ In the subchronic treatment, all male and female mice survived and none showed any toxic symptoms, suggesting good levels of natural tolerance to this preparation. Although the absolute and relative organ weight revealed that EA (0.25–0.50 g/kg) did not cause any organ swelling, atrophy or hypertrophy, the highest dose of EA (1 g/kg) caused a significant increase in the absolute and relative weight of liver in both sexes.

In the current work, hepatic function has been monitored by the evaluation of serum levels of the transaminases AST, ALT and ALP. Biochemical analyses revealed no significant alterations in the serum parameters evaluated in male and female mice, with biomarker values staying within the normal range for the species.^60^, ^61^ Furthermore, we investigated whether the lack of alterations of toxicity indicators in serum was reflected in the histopathological structure of liver tissue, with liver sections being microscopically observed. We observed no significant pathological changes in the liver tissue of EA (1 g/kg)‐treated groups. Moreover, the histological structure was very similar to that of the vehicle‐treated male and female mice. Microscopic evaluation of the lowest doses (0.25 and 0.50 g/kg) was not performed in accordance with health guidelines.^14^ The European Society of Toxicologic Pathology has discussed the significance of hepatomegaly as an adaptive non‐adverse change to a xenobiotic and of little relevance to humans.^62^ Consequently, our results suggest that EA administration does not induce any hepatotoxicity in male and female mice.

Renal function was evaluated by urea and creatinine serum levels as well as the histopathological evaluation of kidneys from mice treated with the vehicle or with EA. The subchronic treatment for all doses of EA did not alter urea and creatinine serum levels in both male and female mice. Moreover, in the histological analysis, no alterations in kidney morphology were observed in animals treated with EA 1 g/kg, suggesting that EA does not have a nephrotoxic action. Finally, the leucocyte differential count showed no treatment‐related differences in neutrophils, monocytes, eosinophils and lymphocytes counts evaluated at any dose used in either sex.

## Conclusion

We here provided the first *in vivo* evidence that EA – a fraction chemically rich in styryl‐2‐pyrones and dihydrostyryl‐2‐pyrones compounds obtained from *P. sabulosa* – does not induce any tolerance in mice to its anxiolytic‐like and anticonvulsant effects after protracted treatment. Our findings also indicate the potentially beneficial effects of EA in counteracting the anxiogenic‐like actions observed during early DZP withdrawal. Additionally, acute and subchronic toxicity assays suggested that EA has low toxicity as it did not cause any deaths or significant changes in the main biochemical, haematological and histopathological markers, being all values within normal physiological limits. Further complementary studies, such as chronic toxicity, mutagenicity and carcinogenicity tests, are now necessary to confirm the safety of medicines derived from this plant. Moreover, it would be desirable to perform toxicological evaluations of EA on a range of non‐rodent species.

## Declaration

### Acknowledgements

This work was supported by CNPq, FACEPE and CAPES which provided research grants to FS Duarte, M Duzzioni and TCM De Lima. The authors are grateful to ‘Fundação José Pedro de Araújo’ (MG, Brazil) for the partial financial support.

### Conflict of interest

No conflict of interest to be declared.
